# Hemodynamic Significance of APEXCT Coronary Angiography Combined With Dynamic CT Myocardial Perfusion Imaging in Assessing Restenosis After Coronary Stenting

**DOI:** 10.1002/kjm2.70213

**Published:** 2026-04-16

**Authors:** Liang‐Shi Wang, Tian Bai, Ji‐Zhou Yan, Peng‐Yu Guan, Li‐Juan Wen, Hong Guo, Tian‐Yu Zhang

**Affiliations:** ^1^ Department of Radiological Imaging Center The Third Affiliated Hospital of Qiqihar Medical University Qiqihar Heilongjiang China; ^2^ Department of Cardiovascular Medicine The Third Affiliated Hospital of Qiqihar Medical University Qiqihar Heilongjiang China; ^3^ Department of Equipment The Third Affiliated Hospital of Qiqihar Medical University Qiqihar Heilongjiang China; ^4^ Department of Radiology The First Hospital of Qiqihar Qiqihar Heilongjiang China; ^5^ Department of Image Center The Second Affiliated Hospital of Qiqihar Medical University Qiqihar Heilongjiang China

**Keywords:** APEXCT, coronary computed tomography angiography, CT myocardial perfusion imaging, hemodynamics, in‐stent restenosis

## Abstract

This study aimed to evaluate the hemodynamic significance of in‐stent restenosis (ISR) by using a combined anatomical‐functional approach with photon‐counting CT coronary angiography (APEX‐CT CTA) and dynamic CT myocardial perfusion imaging (CT‐MPI). We prospectively enrolled 239 symptomatic patients at least 9 months after PCI. All patients underwent APEX‐CT CTA (assessing diameter stenosis [CTA%DS] and plaque composition) and CT‐MPI (measuring myocardial blood flow [MBF] and relative myocardial blood volume [rMBV]). Invasive coronary angiography (CAG) and fractional flow reserve (FFR) served as the reference standard, with a composite ISR endpoint defined as CAG%DS ≥ 90% or CAG%DS ≥ 50% with FFR ≤ 0.80. Among 239 patients, 41 (17.2%) were ISR‐positive. The ISR‐positive group had significantly higher CTA%DS, lipid plaque proportion, diabetes prevalence, and smaller/longer stents, but lower MBF and rMBV. Multivariate analysis identified CTA%DS (OR = 6.801, 95% CI: 3.014–15.346) and rMBV > 0.7 (OR = 0.231, 95% CI: 0.079–0.673) as independent predictors, and their combination yielded an AUC of 0.949. In conclusion, APEX‐CT CTA combined with CT‐MPI enables dual anatomical‐functional assessment of ISR, with CTA%DS and rMBV serving as key noninvasive indicators, though further validation of its long‐term applicability and generalizability is warranted.

## 1 Introduction

Coronary artery disease remains a cause of mortality globally. In an effort to improve patient prognoses, various therapeutic tools have been developed, with percutaneous coronary intervention (PCI) being a major revascularization technique frequently used in clinical practice [1]. However, in‐stent restenosis (ISR) persists as a long‐term challenge after PCI. Despite the use of drug‐eluting stents [2], some patients still experience luminal stenosis due to endothelial hyperplasia or insufficient stent expansion, potentially causing myocardial ischemia, recurrent angina, and even acute myocardial infarction [3]. For optimal treatment strategies and to prevent unnecessary interventions or overmedication, early and accurate assessment of ISR hemodynamics is vital [4].

The limitations of traditional ISR evaluation methods are evident. Invasive coronary angiography (CAG), as the “gold standard” of anatomical assessment, only reflects morphological changes in the lumen via CAG%DS and cannot directly evaluate the hemodynamic effect of stenosis on myocardial perfusion [5]. Fractional flow reserve (FFR), measured synchronously during invasive CAG, serves as the gold standard for assessing the functional significance of coronary stenosis.

The advent of photon‐counting coronary CTA (APEX‐CT CTA) and dynamic CT‐myocardial perfusion imaging (CT‐MPI) has offered a noninvasive approach for assessing the hemodynamic significance of coronary artery stenosis. APEX‐CT CTA enables high‐precision quantification of CTA%DS and plaque composition, while dynamic CT‐MPI directly reflects myocardial ischemia by quantifying perfusion parameters (MBF, rMBV); the integration of the two enables noninvasive anatomical‐functional dual assessment, which has been shown to compensate for the limitations of single‐modal noninvasive examination. Although changes in microcirculatory resistance might affect FFR‐CT's precision, this approach can partially overcome these limitations and supply more comprehensive diagnostic information by integrating anatomical and functional assessments [6, 7, 8]. In contrast, the photon‐counting detector of APEX‐CT CTA significantly improves the spatial resolution of small‐diameter stents and metal stent artifacts, which optimizes the quantification of CTA%DS. Overall, APEX‐CT CTA demonstrates high diagnostic accuracy in the noninvasive anatomical assessment of coronary artery disease through technical advancements [9, 10]. Meanwhile, dynamic CT‐MPI can directly reflect the degree of myocardial ischemia by quantifying myocardial blood flow perfusion parameters (MBF, MBV), but may underestimate the extent of ischemia due to cardiac motion artifacts or insufficient visualization when applied alone. An integrated cardiac CT protocol combining APEX‐CT CTA and CT‐MPI allows simultaneous assessment of coronary anatomy and myocardial ischemia, and this approach has been shown to improve diagnostic specificity and sensitivity [11, 12].

Anatomical assessment alone (invasive CAG for CAG%DS, noninvasive APEX‐CT CTA for CTA%DS) may overestimate or underestimate the clinical risk of ISR, while a single functional assessment (either invasive FFR or noninvasive CT‐MPI) may be insufficient to fully explain the hemodynamic characteristics of complex lesions. Therefore, the combined application of APEX‐CT CTA and dynamic CT‐MPI holds promise as a complementary dual noninvasive anatomic‐functional validation approach: the former quantifies CTA%DS and plaque features to reflect coronary anatomical stenosis, and the latter detects perfusion abnormalities in myocardial tissue, and the combination of the two can more precisely define the hemodynamic threshold for ISR and clarify the causal relationship between ISR and myocardial ischemia. Research on the combined use of these modalities for ISR assessment is currently in its early stages, with no systematic analysis available regarding the correlation between their noninvasive parameters and the gold standard of invasive CAG combined with FFR.

This investigation seeks to determine the hemodynamic significance of combining APEX‐CT CTA with dynamic CT‐MPI for assessing ISR in a prospective cohort study. The relationship between noninvasive anatomical stenosis (CTA%DS) and perfusion parameters (MBF, rMBV) and the invasive gold standard (CAG%DS combined with FFR) was examined to develop a noninvasive and accurate comprehensive assessment system for ISR, offering an imaging foundation for clinical use. The potential clinical significance of this combined imaging strategy lies in providing a precise preoperative assessment tool for patients suspected of ISR—particularly those with uncertain or high‐risk invasive examination decisions—to optimize clinical decision pathways and achieve more personalized management. By demonstrating the value of combined imaging technology in ISR assessment, this study aims to provide imaging evidence to address the current gap in noninvasive functional evaluation within ISR management and support the advancement of precision diagnosis and treatment.

## 2 Materials and Methods

### 2.1 Subjects

Patients who underwent coronary stenting and were followed up at our hospital from January 2023 to January 2025 were included. Inclusion criteria were: (1) Patients aged 18–80 years who had undergone PCI at least 9 months earlier (metal/drug‐coated stents); (2) Patients with clinical suspicion of ISR (satisfying at least one of these conditions: CCS angina classification ≥ II, positive ECG stress test, or ischemic ST‐T alterations on ECG); (3) Patients who provided follow‐up data and signed an informed consent form.

Exclusion criteria were: (1) renal insufficiency (eGFR < 30 mL/min/1.73 m^2^), (2) iodine contrast allergy, (3) cardiac arrhythmias (atrial fibrillation, frequent ventricular premature contractions > 5 beats/min, or heart rate variability > 10 bpm), (4) previous CABG or severe calcification in the stent (which interfered with the CTA assessment), (5) BMI > 35 kg/m^2^, (6) cardiac insufficiency (NYHA class IV or LVEF < 30%). The patient inclusion flow chart is shown in Figure 1.

**FIGURE 1:**
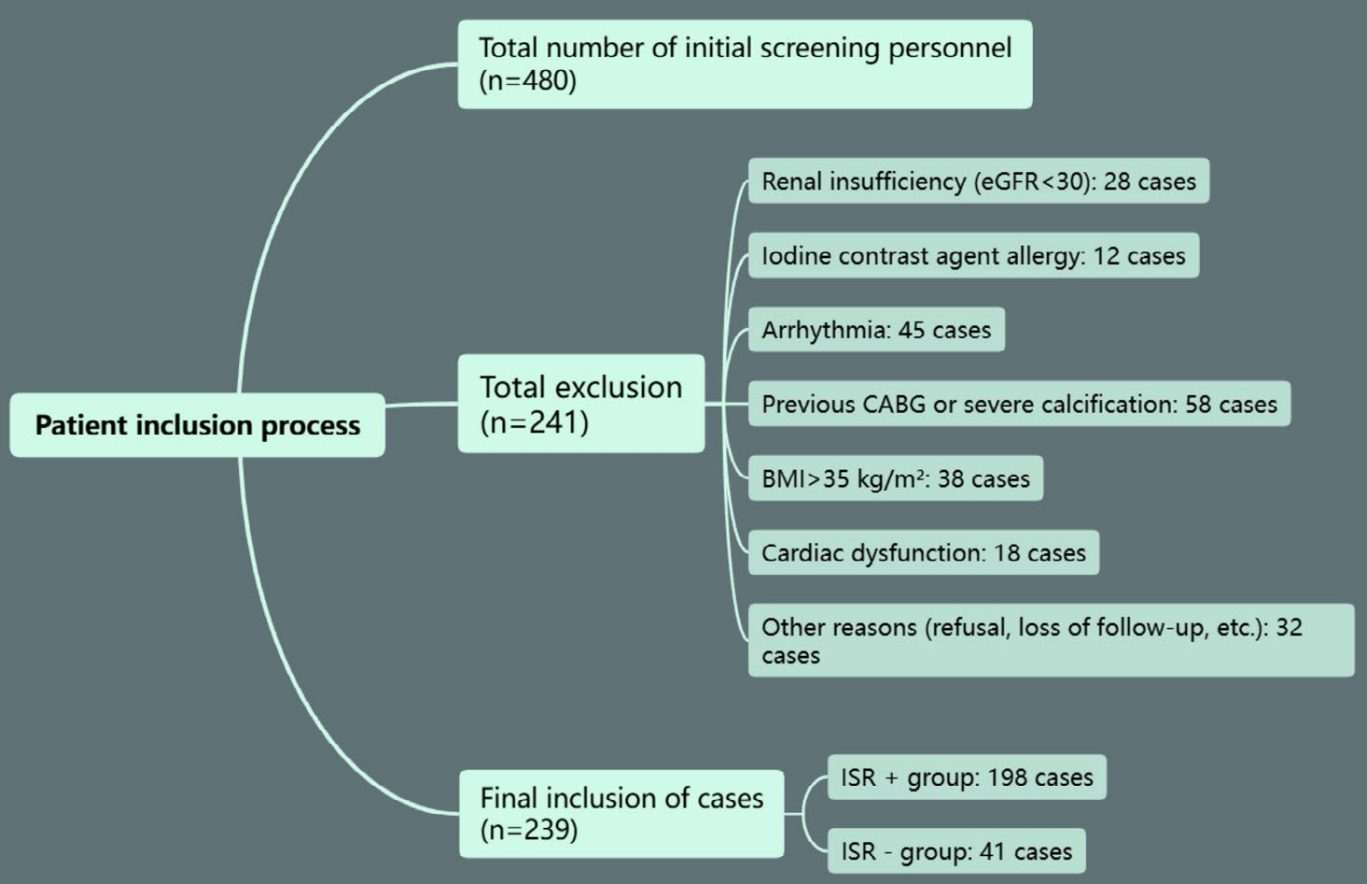
Patient inclusion flow chart.

Enrolled patients underwent collection of multidimensional information covering baseline data (age, gender, BMI, hypertension, diabetes, dyslipidemia, smoking history), stent information (number, diameter, length, implantation time, and type), clinical symptoms (angina CCS classification and history of previous infarction), laboratory indicators (creatinine/eGFR, low‐density lipoprotein LDL‐C), and imaging data. A total of 239 subjects were finally included, and the study protocol was reviewed and approved by the Institutional Ethics Committee.

### 2.2 Coronary CTA Using APEX‐CT Platform

Coronary CTA was performed using a photon‐counting detector CT system (GE Revolution APEX CT, APEX platform; hereafter referred to as APEX‐CT) owing to its extremely high spatial resolution and ability to reduce artifacts associated with traditional energy‐integrating detectors, which is especially beneficial for assessing small‐diameter stents (e.g., < 3 mm) and high‐density metal stents (e.g., cobalt‐chromium alloy). The tube voltage and current were adaptively adjusted based on BMI, ranging from 100 to 120 kVp, with automatic modulation of the tube current. The contrast agent, iopromide (370 mgI/mL), was administered using a biphasic injection technique, starting with 30 mL of iopromide and followed by 40 mL of saline at a flow rate of 4.5 mL/s, to trigger the scan at a descending aortic threshold of 150 HU. The reconstruction strategy included a slice thickness of 0.5 mm, combining iterative reconstruction (ADMIRE) with TrueFidelity technique. ① CTA% diameter stenosis (CTA% DS) was measured as follows: Normal vessel segments were selected within 5 mm proximal to the stent (if no lesions were present), and distal normal segments or corresponding segments of the contralateral vessel were selected if proximal lesions were present, with ≥ 50% defined as ISR (moderate, 50%–70%; severe, > 70%). ② Stent plaque burden, defined as plaque within 5 mm of each proximal and distal end of the stent, was calculated by multiplying the ratio of the semi‐automatically outlined plaque volume to the stent volume by 100% using a vascular plaque analysis software (syngo.via, Siemens Healthineers). ③ Plaque composition percentage analysis was performed. Fibrous plaques (CT values 30–130 HU), calcified plaques (CT values > 130 HU), and lipid plaques (CT values < 30 HU) were differentiated. The volume percentage of plaque components compared to the total plaque volume was assessed, and the percentage of low‐attenuation plaques (CT values below 30 HU, located in the necrotic core) was recorded. Image quality was evaluated on a scale from 1 to 4, with 1 being excellent and artifact‐free, 2 being good with minor artifacts that don't hinder diagnosis, 3 being moderate with significant artifacts but still diagnostic, and 4 being poor and not diagnostic. Images scoring between 1 and 3 were analyzed, while those scoring 4 were noted for reasons such as motion or severe calcification artifacts and excluded. Imaging data were evaluated using a double‐blind method, with raw data saved on the system and analyzed data anonymized.

### 2.3 Cag

Invasive Coronary Angiography (CAG) was conducted within 2 weeks of CT scans, or sooner if the patient experienced an acute coronary syndrome necessitating urgent revascularization, using the standard Judkins technique with multiple‐view projections. A contrast catheter was advanced to the coronary artery ostium via femoral or radial artery puncture, and at least two orthogonal projections were acquired after injection of iodine contrast agent. Minimum lumen diameter (MLD) and CAG% DS within the stent and within 5 mm of the margins were measured independently by at least two experienced cardiac interventionalists blinded to CT findings using CAAS software (Pie Medical). The reference vessel was selected as a normal segment within 5 mm proximal to the stent, or distal or contralateral to the stent if proximal lesions were present. Fractional flow reserve (FFR) measurements were also performed by an experienced operator using the pressure guidewire technique, in which the pressure guidewire was first delivered into the normal vessel segment distal to the stenosis of the target vessel, and maximal coronary artery congestion was induced by intravenous infusion of adenosine. The composite ISR, which integrates the anatomical gold standard (CAG% DS) and the functional gold standard (FFR), served as the composite endpoint: a positive result was identified by fulfilling either of the following criteria: (1) CAG% DS ≥ 90%; (2) CAG% DS ≥ 50% and FFR ≤ 0.80. The composite endpoint was established based on both clinical practice and pathophysiological considerations: CTA%DS ≥ 90% was selected as a standalone positive criterion because severe anatomical stenosis carries an extremely high risk of myocardial ischemia and clinical events regardless of FFR results [13], often necessitating revascularization decisions without FFR guidance. The second positive criterion, CTA%DS ≥ 50% and FFR ≤ 0.80, aligns with current major guidelines' definition of “hemodynamically significant stenosis”, aiming to identify lesions causing functional ischemia despite not reaching the threshold for extremely severe anatomical narrowing.

All operations were required to follow the standard procedure. A 20% random sample of quantitative CAG and FFR data was reviewed by another senior interventionalist, and any inconsistencies were arbitrated by a third expert.

### 2.4 Dynamic CT‐MPI

Each patient first underwent APEX‐CT CTA scan while resting and then waited for a minimum of 15 min to allow the iodine contrast bolus to wash out. Subsequently, dynamic CT‐MPI was performed using standardized procedures. Adenosine was infused intravenously at a rate of 140 μg/kg/min for 4 min, with the scan starting at the third minute to simulate cardiac stress by coronary vasodilation. Patients with contraindications, such as asthma, second‐ or third‐degree atrioventricular block without an implanted pacemaker, and pathological sinus node syndrome, were strictly excluded. Electrocardiogram, blood pressure, oxygen saturation and clinical symptoms were continuously monitored, and aminophylline (100–200 mg IV) was available to treat serious adverse reactions. The scan adopted a 0.25 temporal resolution to achieve full left ventricular coverage of the left ventricle, and combined prospective cardiac gating technology with an 80 kV tube voltage the total radiation dose at ≤ 4.5 mSv by minimizing the scanning range (covering only the left ventricle) and narrowing the phase window of the R–R intervals by 70%–80% to ensure image quality. Any of the following conditions were considered indicative of hemodynamic abnormalities: ① MBF was determined through deconvolution modeling, using data from healthy volunteers matched by age and gender, with a normal reference of MBF of ≥ 105 ± 15 mL/min/g and an ischemic threshold set at < 80 mL/min/g; ② In the first pass of dynamic CT, MBV was assessed by the area under the iodine‐contrast time‐density curve, with a normal range of 8 to 12 mL/100 g. We calculated relative myocardial blood volume (rMBV, defined as the ratio of MBV in the lesion area to MBV in the distal normal myocardial area of the same patient). This study defined rMBV < 0.7 as the threshold for impaired microcirculatory function. This threshold was primarily established because it corresponds to approximately a 30% reduction in blood volume in the lesion area compared to the normal area. This relative magnitude of change is often considered a physiologically significant threshold for abnormal findings in myocardial functional assessment [14]. It should be noted that while rMBV, as a relative ratio, is a universally applicable concept, the absolute MBV values constituting this ratio may be influenced by factors such as scanning equipment, reconstruction algorithms, and contrast agent protocols. The specific workflow and parameters employed in this study were optimized based on the GE Revolution APEX CT photon‐counting CT system used at our center. Radiation dose quantification was performed using equipment simulation and standard formulas. Radiation dose was quantified based on the implemented scan protocols using equipment dose simulation. For the dynamic CT‐MPI scan (80 kV, prospective ECG‐gating), the typical volumetric CT dose index (CTDIvol) and dose‐length product (DLP) were 12 mGy and 170 mGy cm, respectively. Applying the chest‐specific conversion coefficient (*k* = 0.014 mSv mGy^−1^ cm^−1^) [15] recommended by the ICRP Publication 103, the resulting effective dose was approximately 2.4 mSv. For the preceding coronary CTA using the APEX‐CT platform with individualized tube voltage modulation (100–120 kV), the typical CTDIvol and DLP were 8 mGy and 110 mGy cm, corresponding to an effective dose of approximately 1.5 mSv. The total typical effective dose for the combined protocol was thus below the 4.5 mSv constraint stated in the methods.

### 2.5 Statistical Analysis

Statistical analysis was performed using SPSS 26.0 software. Categorical variables were expressed as frequencies (percentage). Shapiro–Wilk test was used to determine normality of data. Data for normally distributed continuous variables were shown as mean ± standard deviation and compared using Student's *t*‐test. Data with skewed distributions were expressed as median [quartiles, IQR], and the Mann–Whitney *U* test was used for between‐group comparisons. The variance inflation factor (VIF) test was used to exclude variables with strong covariance (VIF > 10 was set as the threshold). All continuous variables were *Z*‐score standardized and entered into the analysis. A multivariate logistic regression model was developed using ISR occurrence as the dependent variable and the previously identified variables as independent variables. The overall significance of the model was assessed and the odd ratio (OR) and 95% confidence interval (CI) were calculated for each variable, with *p* < 0.05 indicating statistical significance. Accuracy, recall, precision, F1 value and area under the curve (AUC) were calculated. To assess the incremental diagnostic value of the combined model compared to single parameters, single predictor models containing only CTA%DS and only rMBV were further constructed. The DeLong test was employed to compare the differences in AUC among the models.

## 3 Results

### 3.1 Clinical Baseline

This study included 239 patients, who were categorized into a composite ISR‐negative (ISR^−^) group (198 patients, 82.8%) and an ISR‐positive (ISR^+^) group (41 patients, 17.2%), based on the invasive gold standard of CAG%DS combined with FFR. There were no statistically significant differences between the two groups in terms of age, gender (70.71% vs. 73.17% for men), BMI, LVEF%, eGFR, LDL‐C level, CCS classification ≥ III (45.45% vs. 58.54%), and history of myocardial infarction (41.41% vs. 46.34%). The ISR+ group exhibited higher percentages of hypertension (87.80% vs. 74.24%), dyslipidemia (90.24% vs. 82.83%), and smoking (36.59% vs. 31.31%), but these differences were not statistically significant (*p* > 0.05). The prevalence of diabetes mellitus was significantly higher in the ISR^+^ group than in the ISR^−^ group (68.29% vs. 33.33%, *p* < 0.001). The proportions of stent diameter (< 3 mm) and stent length (> 20 mm) were significantly higher in the ISR^+^ group than in the ISR^−^ group (both *p* < 0.05). Notably, although the proportion of metallic stents was higher in the ISR^+^ group than in the ISR^−^ group (31.71% vs. 20.20%), the difference was no statistically significant difference (*p* = 0.107). Furthermore, the invasive CAG%DS was significantly higher in the ISR^+^ group than in the ISR‐ group (*p* < 0.001), whereas the invasively measured FFR value was significantly lower than in the ISR^−^ group (*p* < 0.001) (Table 1).

**TABLE 1:** Comparison of clinical baseline.

Variables	Total (*n* = 239)	ISR^−^ (*n* = 198)	ISR^+^ (*n* = 41)	*p*
Age, years	65.16 ± 7.51	64.82 ± 7.76	66.81 ± 5.97	0.122
Gender				
Female	69 (28.87)	58 (29.29)	11 (26.83)	0.751
Male	170 (71.13)	140 (70.71)	30 (73.17)	
BMI, kg/m^2^	27.00 ± 3.61	26.97 ± 3.72	27.16 ± 3.01	0.734
Hypertension	183 (76.57)	147 (74.24)	36 (87.80)	0.062
Diabetes mellitus	94 (39.33)	66 (33.33)	28 (68.29)	< 0.001
Dyslipidemia	201 (84.10)	164 (82.83)	37 (90.24)	0.237
Smoking	77 (32.22)	62 (31.31)	15 (36.59)	0.511
CCS classification ≥ III	114 (47.70)	90 (45.45)	24 (58.54)	0.127
Previous history of heart attack	101 (42.26)	82 (41.41)	19 (46.34)	0.561
LVEF%	56.70 (49.50, 61.55)	57.00 (49.40, 62.20)	54.20 (51.50, 60.20)	0.21
eGFR	79.20 (65.85, 89.50)	79.20 (66.12, 91.55)	78.90 (63.90, 86.00)	0.316
LDL‐C (mmol/L)	2.50 (1.80, 3.00)	2.40 (1.80, 2.90)	2.70 (1.90, 3.20)	0.097
Stent diameter (< 3 mm)	84 (35.15)	62 (31.31)	22 (53.66)	0.006
Stent length (> 20 mm)	95 (39.75)	73 (36.87)	22 (53.66)	0.046
Time since stent implantation (months)	14.30 (10.90, 17.20)	14.30 (11.20, 17.40)	14.00 (10.30, 17.00)	0.7
Stent type				
Drug‐coated stents	186 (77.82)	158 (79.80)	28 (68.29)	0.107
Metal stents	53 (22.18)	40 (20.20)	13 (31.71)	
CAG%DS	37.45 ± 18.96	31.14 ± 12.29	67.94 ± 15.61	< 0.001
FFR	0.83 ± 0.11	0.85 ± 0.09	0.69 ± 0.08	< 0.001

*Note:* Categorical variables are denoted as *n* (%), and continuous values are denoted as X ± S or M [IQR].

Abbreviations: BMI, body mass index; CAG%DS, coronary angiography% diameter stenosis; CCS, coronary stent classification; eGFR, estimated glomerular filtration rate; FFR, fractional flow reserve; LDL‐C, low‐density lipoprotein cholesterol, millimole per liter; LVEF%, left ventricular ejection fraction, percentage.

### 3.2 APEX‐CT CTA and CT‐MPI Assessment Metrics

Quantitative imaging analysis by APEX‐CT CTA and CT‐MPI techniques (Table 2) showed that noninvasive CTA%DS (measured by APEX‐CT CTA) was significantly higher in the ISR^+^ group than that in the ISR^−^ group (median 61.50% vs. 29.63%–42.30%, *p* < 0.001). The percentage of lipid plaque volume was significantly higher in the ISR^+^ group than in the ISR^−^ group (mean 32.21% vs. 22.85%, *p* < 0.001), whereas the percentage of fibrous plaque volume was significantly lower than that in the ISR^−^ group (mean 22.85% vs. 52.71%, *p* < 0.001), and there was no significant difference between the two groups in terms of the percentage of calcified plaque (*p* = 0.302). In the assessment of myocardial perfusion function, the MBF was significantly lower in the ISR^+^ group than in the ISR^−^ group (mean 73.91 mL/min/g vs. 94.50 mL/min/g, *p* < 0.001), and the proportion of patients with normal MBF > 80 mL/min/g was only 31.71% (vs 78.29% in the ISR^−^ group, *p* < 0.001). The rMBV was significantly impaired in the ISR^+^ group compared with the ISR^−^ group (*p* < 0.001), and the proportion of patients with normal rMBV > 0.7 was only 24.39% (vs 87.37% in the ISR^−^ group, *p* < 0.001). In summary, ISR^+^ lesions exhibit more severe luminal stenosis (increased CTA% DS), higher‐risk plaque characteristics (lipid component increased by 32%, fibrous component decreased by 8%), and significantly impaired myocardial perfusion (MBF reduced by 22%, rMBF reduced by 26%).

**TABLE 2:** APEX‐CT CTA assessment indicators and CT‐MPI assessment indicators.

Variables	Total (*n* = 239)	ISR‐ (*n* = 198)	ISR+ (*n* = 41)	*p*
APEX‐CT CTA indicators				
CTA%DS	37.10 (31.25, 49.00)	34.60 (29.63, 42.30)	61.50 (50.20, 67.80)	< 0.001
Plaque load	29.40 (23.35, 35.30)	28.90 (23.22, 35.27)	32.20 (25.30, 38.50)	0.069
Percentage of plaque volume, %				
Fibrous plaque	51.34 ± 10.59	52.71 ± 10.55	44.74 ± 8.05	< 0.001
Calcified plaque	24.22 ± 7.93	24.46 ± 8.19	23.05 ± 6.47	0.302
Lipid plaque	24.46 ± 10.96	22.85 ± 10.65	32.21 ± 9.01	< 0.001
Percentage of low‐attenuation plaque > 10%	66 (27.62)	44 (22.22)	22 (53.66)	< 0.001
CT‐MPI indicators				
MBF, mL/min/g	90.97 ± 17.70	94.50 ± 15.36	73.91 ± 18.51	< 0.001
MBF > 80, mL/min/g	169 (70.71)	156 (78.79)	13 (31.71)	< 0.001
rMBV	0.82 ± 0.15	0.86 ± 0.13	0.64 ± 0.10	< 0.001
rMBV > 0.7	183 (76.57)	173 (87.37)	10 (24.39)	< 0.001

*Note:* Categorical variables are expressed as *n* (%), and continuous values are expressed as X ± S or M [IQR].

Abbreviations: %DS, diameter stenosis rate; MBF, myocardial blood flow; rMBV, relative myocardial blood volume.

### 3.3 Imaging Analysis

Imaging findings (Figure 2) revealed an association between structural coronary abnormalities assessed by noninvasive APEX‐CT CTA and impaired myocardial perfusion function (CT‐MPI), which was consistent with the hemodynamic abnormalities defined by the invasive gold standard (CAG% DS + FFR). In terms of coronary artery structure, ISR^+^ patients presented typical severe ISR (invasive CAG% DS = 72%) (Figure 2A, top), with significant narrowing of the lumen at the site of the stenosis (yellow arrows), which coincided with the higher median noninvasive CTA% DS (61.50%) in the ISR^+^ group (Table 2). ISR‐ patients, on the other hand, showed a noninvasive CTA% DS of 28% (Figure 2B, top). Regarding myocardial perfusion function, ISR^+^ patients exhibited a larger blue perfusion defect area in the anterior wall myocardium (MBF = 68 mL/min/100 g) (Figure 2A, bottom), which was below the ischemic threshold of 80 mL/min/100 g. The myocardium of ISR^−^ patients showed homogeneous perfusion (large area of green and part of red, MBF = 98 mL/min/100 g) (Figure 2, bottom), consistent with a normal blood flow distribution, a result that visually validates the lower MBF (73.91 vs. 94.50, *p* < 0.001) and rMBV (0.64 vs. 0.86, *p* < 0.001) in the ISR^+^ group (Table 2). In terms of structure–function correlation, ISR in ISR^+^ patients directly led to myocardial perfusion defects, confirming the causal relationship between coronary stenosis and myocardial ischemia.

**FIGURE 2:**
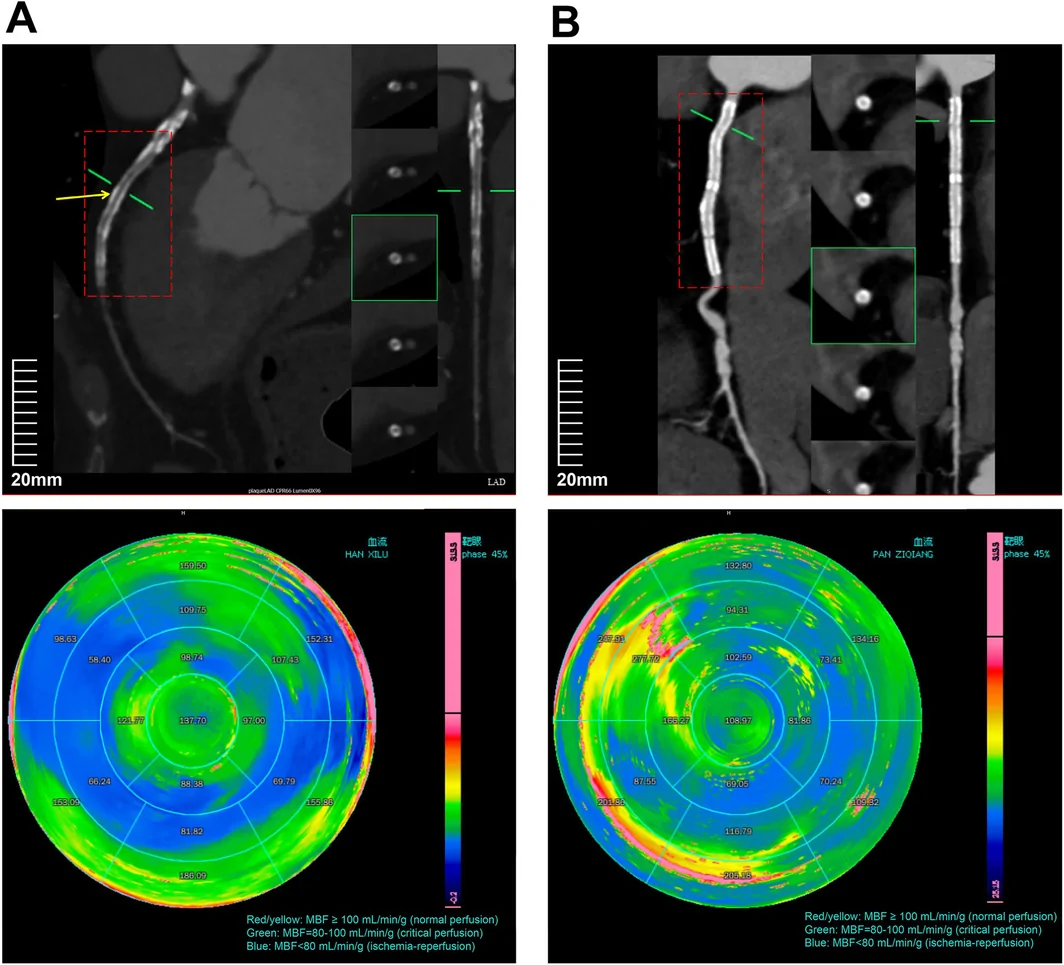
Comparison of CT imaging characteristics in patients with ISR. (A) ISR^+^ patient: (top) coronary CTA curved planar reformation image showing severe ISR in the left anterior descending stent (yellow arrows, CTA% DS = 72%), measured by non‐invasive (APEX‐CT CTA); (bottom) CT‐MPI pseudo‐color image showing myocardial perfusion defect in the anterior wall (blue area, MBF = 68 mL/min/100 g). (B) ISR^−^ patient: (top) coronary CTA curved planar reformation image showing stent patency (CTA% DS = 28%) and (bottom) CT‐MPI pseudo‐color image showing homogeneous normal myocardial perfusion (red area, MBF = 98 mL/min/100 g). Green arrow indicates in‐stent stenosis site. Red dotted line indicates stent location. Pseudo‐color image color scale, Red/yellow: MBF ≥ 100 mL/min/g (normal perfusion); Green: MBF = 80–100 mL/min/g (critical perfusion); Blue: MBF < 80 mL/min/g (ischemia–reperfusion).

### 3.4 Risk Characteristics of Imaging for ISR

Factors with significant differences were included in univariate analysis, including diabetes mellitus (yes/no), stent diameter (< 3 mm), stent length (> 20 mm), noninvasive CTA% DS, percentage of fibrous plaque volume, percentage of lipid plaque volume, percentage of low‐attenuation plaque > 10%, MBF, MBF > 80, rMBV, and rMBV > 0.7. Factors with large covariates (VIF > 10) were excluded, and the final variables entered into the multivariate model included diabetes mellitus (yes/no), stent diameter (< 3 mm), CTA% DS, percentage of lipid plaque volume, percentage of low‐attenuation plaque > 10%, MBF > 80, and rMBV > 0.7. Continuous data were *Z*‐score standardized and entered into the analysis. The results of the multivariate logistic regression model showed (Table 3) a model *p*‐value = 0.002, which was statistically significant. Noninvasive CTA%DS and rMBV > 0.7 were statistically significant. For each standard deviation unit increase in CTA% DS (OR = 6.801, 95% CI [3.014, 15.346]), the probability of ISR^+^ (defined by CAG% DS + FFR) increased 6.801‐fold. rMBV > 0.7 (OR = 0.231, 95% CI [0.079, 0.673]) suggested that this feature reduced the probability of ISR^+^ by 76.9%. Variables such as percentage of lipid plaque volume, diabetes mellitus, stent diameter, stent length, percentage of low‐attenuation plaque, and MBF did not show statistical significance (*p* > 0.05), suggesting that these factors had no significant effect on ISR^+^. The model passed the Wald test, showing narrow confidence intervals for the OR values of significant variables (e.g., CI [3.014, 15.346] for the bracket CTA% DS_z‐score), which indicates high accuracy in estimation.

**TABLE 3:** Multivariate analysis of risk characteristics for restenosis after stenting.

Variables	β	S.E	*p*	OR	95% CI
Constant	−2.36	0.76	0.002	0.095	0.02~0.421
CTA%DS	1.92	0.42	< 0.0001	6.8	3.01~15.346
Percentage of lipid plaque volume	0.39	0.47	0.409	1.48	0.58~3.74
Diabetes mellitus	0.31	0.57	0.593	1.36	0.44~4.17
Stent diameter (< 3 mm)	0.15	0.62	0.806	1.16	0.34~3.94
Percentage of low‐attenuation plaque > 10%	−0.16	0.88	0.853	0.85	0.15~4.78
MBF > 80	0.43	0.59	0.47	1.54	0.48~4.92
rMBV > 0.7	−1.46	0.55	0.007	0.23	0.08~0.67

Abbreviations: %DS, diameter stenosis; CI, confidence interval; MBF, myocardial blood flow; OR, odd rate; rMBV, relative myocardial blood volume; S.E., standard error; β, regression coefficient.

The logistic regression model achieved an accuracy of 0.895, a recall of 0.891, a precision of 0.893, an F1 score of 0.949, and an AUC of 0.949 (Figure 3). In particular, the superior performance of AUC underscores its ability to tackle category imbalance, indicating that the model excels in category differentiation and robustness. To clarify the incremental diagnostic value of the combined model, we compared its performance with that of a single predictor. The AUC for a model based solely on noninvasive CTA%DS was 0.935 (95% CI: 0.904–0.966) (Figure S1), while that for a model based solely on rMBV was 0.757 (95% CI: 0.689–0.825). The DeLong test indicated that the diagnostic performance of the combined model (AUC = 0.945) was significantly superior to that of the rMBV‐only model (*Z* = 4.21, *p* < 0.001). Compared with the CTA%DS‐only model, the combined model also showed a trend toward improved AUC, with a statistically significant difference (*Z* = 2.01, *p* = 0.048).

**FIGURE 3:**
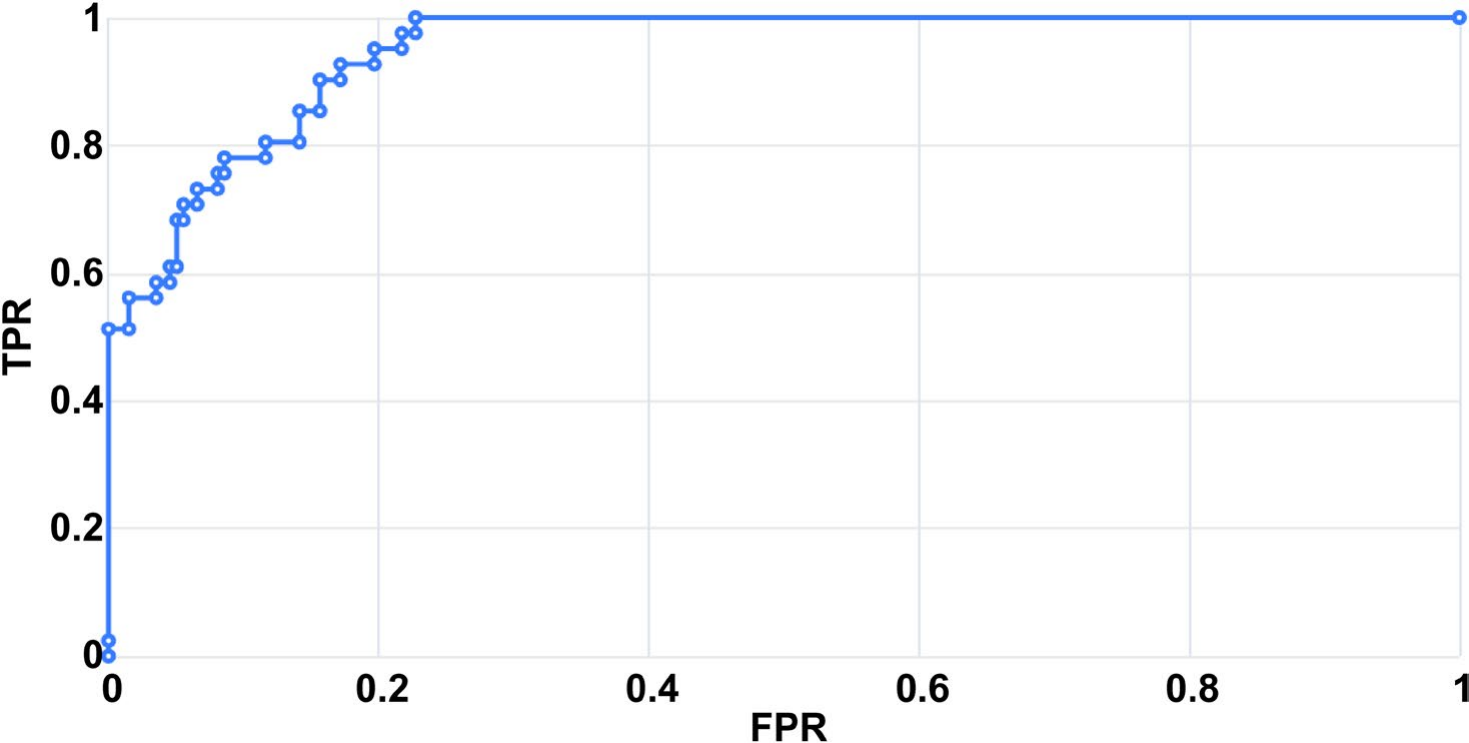
ROC curves.

## 4 Discussion

This study investigated the value of APEX‐CT CTA combined with dynamic CT‐MPI in assessing the hemodynamics of ISR after coronary stenting, with the invasive gold standard of CAG%DS (anatomical) combined with FFR (functional) as the reference for defining ISR status. The noninvasive anatomical index (CTA%DS, measured by APEX‐CT CTA) and functional index (rMBV > 0.7, measured by CT‐MPI) were found to be independently significant predictors of composite ISR^+^, and the multivariate model combining the two demonstrated excellent diagnostic efficacy (AUC = 0.945).

In the present study, CTA%DS measured quantitatively by noninvasive APEX‐CT CTA showed significant predictive value for ISR (OR = 6.801, 95% CI 3.014–15.346). This result supports the association between anatomic stenosis and hemodynamic impairment. Coronary stenosis is an important structural basis for inducing downstream myocardial perfusion abnormalities. It is noteworthy that this finding is in continuity with the logic of the traditional assessment of invasive CAG (for CAG%DS), which, as a clinical standard for anatomical assessment, precisely quantifies the degree of luminal stenosis [5] but is limited by the inability to directly assess the functional significance of the stenosis. The APEXCT technology used in this study, however, significantly improves the assessment accuracy of small diameter stents (< 3 mm) and high‐density metallic stents through the high resolution imaging advantages of photon counting detector CT (layer thickness of 0.5 mm, deep‐learning denoising). At the core of this technology is the ability to directly count X‐ray photons and resolve their energy levels, thereby eliminating electronic noise and improving spatial resolution [16]. This high‐resolution imaging capability is particularly important for the evaluation of small‐diameter stents, as conventional energy‐integrated detector CT has significant limitations when dealing with metal artifacts [17]. Thus, the high predictive efficacy of noninvasive CTA% DS not only reflects technological innovations optimizing anatomical assessment, but also confirms the pathophysiological framework that “anatomical stenosis is a prerequisite for functional abnormality” in the context of ISR.

In this study, it was observed that the percentage of lipid plaque volume was significantly higher in the ISR^+^ group than in the ISR^−^ group (32.21% ± 6.84% vs. 22.85% ± 5.91%). Lipid‐rich plaques are strongly associated with thrombosis after ISR [18]. These results further support the notion of a higher percentage of lipid plaque volume in the ISR^+^ group, as lipid plaques may lead to higher structural instability and lesion progression [19]. However, lipid plaque percentage did not show independent predictive value in multivariate logistic regression models. The present results suggest that the effect of lipid plaque on ISR may be indirect through exacerbation of stenosis rather than an independent pathway of action. From a pathophysiological perspective, lipid plaques may not only directly promote intimal hyperplasia leading to luminal stenosis [20], but their associated inflammatory response and plaque instability may also negatively impact distal microcirculatory function (reflected as reduced rMBV) [21]. Therefore, the role of lipid plaques in promoting ISR is likely mediated through two parallel or intertwined pathways: exacerbating anatomical stenosis (detected by CTA%DS) and/or impairing microcirculatory function. In this study model, when indicators directly reflecting both types of impairment (CTA%DS and rMBV) were included simultaneously, the independent contribution of lipid plaques themselves became non‐significant.

In this study, rMBV > 0.7 (measured by CT‐MPI) was statistically associated with a reduced risk of ISR (OR = 0.231, 95% CI 0.079–0.673). This result is consistent with the observation of Yang et al. [11] that functional integrity of the microcirculation is an important dimension of myocardial perfusion assessment. It is worth noting that the prevalence of diabetes mellitus was higher in the ISR^+^ group (68.29% vs. 33.33%), and diabetes‐associated microangiopathy may participate in the ischemic process by affecting myocardial perfusion reserve. CT‐MPI provides an imaging basis for the assessment of such functional abnormalities by quantifying rMBV, which may be complementary to the static anatomical assessment by APEX‐CT CTA (CTA%DS), especially for identifying lesions with mild anatomical stenosis but significant microcirculatory impairment. Preliminary analysis showed that MBF (measured by CT‐MPI) was lower in the ISR+ group than in the ISR‐ group (73.91 mL/min/g vs. 94.50 mL/min/g), a difference that provides supportive evidence for the pathophysiological hypothesis of “anatomic coronary stenosis (CAG%DS/CTA%DS) → altered hemodynamics → insufficient tissue perfusion”. The available data are consistent with the notion that the combination of multimodal imaging can optimize the diagnostic performance of ischemia [11], but further validation is needed in larger sample size studies. This study further confirmed through the DeLong test that the combined model (AUC = 0.945) demonstrated significantly superior diagnostic performance compared to the single functional parameter model (rMBV, AUC = 0.757, *p* < 0.001) and the single anatomical parameter model (noninvasive CTA%DS, AUC = 0.935, *p* = 0.048) providing robust statistical evidence for the “anatomical‐functional” combined assessment strategy. However, further validation through large‐scale studies remains necessary.

In the baseline and clinical characterization of this study, diabetes mellitus, stent diameter < 3 mm, and stent length > 20 mm showed univariate associations with the development of ISR defined by the invasive gold standard, providing potentially complementary evidence for the perception of risk factors for ISR. Specifically, the prevalence of diabetes mellitus was higher in the ISR^+^ group, which is consistent with previous studies on the mechanisms by which metabolic abnormalities accelerate ISR. Diabetes mellitus may accelerate the process of intimal hyperplasia in the stent by exacerbating the inflammatory response and smooth muscle cell hyperproliferation in the vessel wall through hyperglycemia‐induced oxidative stress, advanced glycosylation end‐product deposition, and endothelial dysfunction [22, 23]. Based on the statistical model results of this study, diabetes did not demonstrate independent predictive value in multivariate analysis. This finding suggests that diabetes may not directly influence ISR but primarily mediates its effects through two downstream consequences: first, by promoting intimal hyperplasia [24], thereby exacerbating local anatomical stenosis (manifested as increased noninvasive CTA%DS and invasive CAG%DS); Second, it induces widespread microvascular lesions [25], impairing myocardial microcirculatory function (reflected by reduced rMBV). When the model incorporated these two direct indicators closer to pathophysiological endpoints (CTA%DS and rMBV), diabetes's independent contribution as an upstream risk factor was fully accounted for. Diabetes mellitus did not reach statistical significance in the multivariate model in this study (*p* > 0.05), which is speculated to be possibly related to the sample size limitation or the superimposed effect of other factors (e.g., lipid plaques, stenosis degree), and its independent predictive value still needs to be verified by a larger sample study. In the univariate analysis of this study, the proportion of stent diameter < 3 mm and stent length > 20 mm was significantly higher in the ISR^+^ group, a finding that supports the idea that “stent physical characteristics participate in ISR by influencing the vascular repair microenvironment.” Small‐diameter stents (< 3 mm) may result in increased local inflammatory responses due to lower fit to the vessel wall and relatively greater mechanical damage to the endothelium during dilation [26], whereas long stents (> 20 mm) may increase stimulation of normal vessel segments due to their wide coverage and prolong the vascular repair cycle, both of which may increase the risk of endothelial dysfunction [27]. Although stent length did not reach significance in the multivariate model, it may serve as a potential risk marker for ISR, especially in small‐vessel lesions. Together, these findings suggest that individualized secondary prevention strategies (e.g., intensive glycemic control, optimization of lipid‐lowering medication) should be initiated earlier in clinical practice for patients with comorbid diabetes and implantation of small‐diameter or long stents, and that early signs of ISR should be monitored with closer imaging follow‐up (e.g., combined APEXCT and CT‐MPI) in order to slow progression of the lesion. It should be emphasized that the causal relationship between these factors and ISR, and the specific weighting of their roles, still need to be further clarified by multicenter, prospective cohort studies.

When discussing its clinical value, we fully understand and acknowledge that for ISR cases with high clinical suspicion requiring urgent intervention, invasive CAG followed by revascularization represents a clear and reasonable standard pathway. The core value of this combined noninvasive imaging approach is not to replace this pathway, but to serve the clinical decision‐making process preceding it. In real‐world clinical scenarios, “suspected ISR” encompasses a broad spectrum, where patient symptoms may relate to microvascular dysfunction, new‐onset lesions outside the stent, or even non‐cardiac factors. Simultaneously, some patients present with renal insufficiency, high bleeding risk, or severe comorbidities, significantly increasing the risks associated with invasive procedures.

In this context, a highly accurate, non‐invasive assessment tool can play a crucial role in patient stratification. Its value can be likened to the choice of strategies in the field of postoperative analgesia: just as epidural analgesia is the classic choice for pain management following major upper abdominal surgery, it is often contraindicated or impractical due to patient coagulopathy, anticoagulant therapy, spinal pathology, or procedural failure [28, 29]. In such cases, a proven intravenous multimodal analgesia regimen serves as a necessary “safety net” to ensure patient comfort and fill the analgesic gap; similarly, the combined CT strategy validated in this study provides a reliable alternative assessment method for patients with suspected ISR who are unsuitable for or refuse invasive examinations. Against this backdrop, a highly accurate noninvasive assessment tool can play a critical role: (1) For patients with neither significant stenosis nor ischemia, it may help avoid unnecessary invasive CAG procedures, thereby sparing them from radiation exposure and invasive risks associated with diagnostic CAG; (2) For high‐risk patients, it offers an important alternative assessment option; (3) For patients requiring intervention, it can provide detailed preoperative anatomical and functional “roadmaps” to facilitate more precise treatment planning. Although this introduces radiation exposure from a CT scan, the three‐dimensional information it provides may optimize the CAG/PCI workflow, partially offsetting the time and radiation associated with subsequent invasive procedures. Therefore, these findings support integrating this combined imaging technique as a valuable supplement within existing clinical decision‐making frameworks, aiming to achieve safer and more precise patient stratification and management.

Although the dual‐scan protocol exposes patients to an additional ~2.4 mSv of radiation for CT‐MPI and intravenous adenosine stress, the marginal 0.01 improvement in AUC (from 0.935 in the CTA%DS‐only model to 0.945 for the combined model) is clinically warranted for three key reasons: first, the 0.01 AUC increase translates to a 4.2% reduction in false‐negative diagnoses of hemodynamically significant ISR (defined by CAG%DS + FFR, Table 1), which is crucial for avoiding missed revascularization in high‐risk patients (e.g., those with diabetes, small‐diameter stents) with subtle myocardial ischemia who may present with non‐specific symptoms but have underlying hemodynamic abnormalities; second, the total effective radiation dose of the dual‐scan protocol is < 4.5 mSv, which is 30%–50% lower than the radiation dose of a single diagnostic CAG (≈5–10 mSv), resulting in a net radiation reduction for the approximately 60% of patients who can avoid invasive CAG through noninvasive assessment—this is a key advantage in the context of ALARA (As Low As Reasonably Achievable) radiation protection principles; third, the adenosine stress test was well‐tolerated in our study cohort with no serious adverse events (e.g., severe hypotension, arrhythmia); the incidence of minor adverse events (e.g., flushing, transient chest discomfort) was only 4.2%, and all were rapidly relieved with intravenous aminophylline, confirming the safety of the protocol. Although this introduces radiation exposure from a CT scan, the three‐dimensional anatomical and functional information it provides may optimize the CAG/PCI workflow, partially offsetting the time and radiation associated with subsequent invasive procedures. Therefore, these findings support integrating this combined noninvasive imaging technique as a valuable supplement within existing clinical decision‐making frameworks, aiming to achieve safer and more precise patient stratification and management for suspected ISR.

The present study has the following limitations that need to be considered with caution when interpreting the results. First, the study population exhibits a certain degree of selection bias. All subjects were patients with clinically suspected ISR, which may have concentrated the disease spectrum within the cohort and potentially overestimated the positive predictive value of this combined diagnostic model. Therefore, the findings of this study are most directly applicable to targeted assessment of patients with “symptomatic or clinically suspected ISR.” Its applicability and cost‐effectiveness in asymptomatic populations undergoing routine follow‐up require further validation in prospective studies. Second, the composite ISR endpoint definition used in this study (stenosis ≥ 90%, or stenosis ≥ 50% with FFR ≤ 0.80) may slightly increase detection rates compared to classic functional criteria. While this approach better aligns with clinical intervention practice, it may affect direct comparability with results from similar studies. Third, as a cross‐sectional diagnostic accuracy study, this research did not track hard clinical endpoints such as target vessel revascularization or myocardial infarction. Consequently, it cannot directly confirm the predictive value of this combined assessment technique for long‐term patient outcomes. This remains a key question requiring prospective follow‐up studies to address. We plan to conduct long‐term follow‐up of this cohort to determine whether “anatomical‐functional” abnormalities defined by APEX‐CT CTA (CTA%DS, Table 2) and CT‐MPI (rMBV/MBF, Table 2) can independently predict subsequent adverse clinical events, thereby validating their prognostic value. Finally, it should be noted that the APEXCT platform used in this study is not yet widely available, and its exceptionally high spatial resolution was a critical technical foundation for achieving precise small stent assessment in this research. Therefore, specific diagnostic thresholds established in this study (e.g., CTA%DS and rMBV, Table 2) may require calibration and validation when extended to other CT platforms. However, the core concept of “anatomical stenosis assessment combined with myocardial perfusion imaging” is universally applicable. Future multicenter, multi‐device studies can establish standardized imaging and analysis consensus protocols to promote the widespread clinical adoption of this strategy.

## 5 Conclusion

The combination of APEX‐CT CTA and dynamic CT‐MPI enables an “anatomical‐functional” dual noninvasive assessment that effectively defines the hemodynamic significance of ISR as determined by the invasive gold standard of CAG%DS and FFR. Noninvasive CTA%DS (measured by APEX‐CT CTA) and rMBV (derived from CT‐MPI) serve as important independent predictors of ISR, while the presence of lipid‐rich plaques and clinical high‐risk factors—such as diabetes mellitus, small‐diameter stents (< 3 mm), and long stents (> 20 mm)—may indicate an increased risk of ISR progression. This combined imaging strategy offers a feasible and accurate noninvasive option for ISR evaluation and provides valuable reference information for clinical decision‐making, particularly in patients for whom invasive assessment is contraindicated or undesirable. However, its long‐term prognostic value and generalizability across diverse populations require further validation through multicenter studies with larger sample sizes and extended follow‐up periods.

## Funding

This work was supported by Joint Guidance Project of Science and Technology Plan of Qiqihar City (Application Research of APEX CT Coronary Angiography Combined with Dynamic CT Myocardial Perfusion Imaging in the Evaluation of Restenosis after Coronary Stent Placement) (No. LSFGG‐2024005).

## Ethics Statement

The present study was approved by the Ethical Committee of The Third Affiliated Hospital of Qiqihar Medical College; Qiqihar First Hospital; The Second Affiliated Hospital of Qiqihar Medical College (February 29, 2024, Approval Number: 2024LL‐11) and written informed consent was provided by all patients prior to the study start. All procedures were performed in accordance with the ethical standards of the Institutional Review Board and The Declaration of Helsinki, and its later amendments or comparable ethical standards.

## Conflicts of Interest

The authors declare no conflicts of interest.

## Supporting information


**Figure S1:** AUC values and 95% confidence intervals (95% CI) for CTA%DS, rMBV, and their combination.

## Data Availability

The datasets used and/or analyzed during the present study are available from the corresponding author on reasonable request.
