# Supplementary figures and images for: Hemodynamic Significance of APEXCT Coronary Angiography Combined With Dynamic CT Myocardial Perfusion Imaging in Assessing Restenosis After Coronary Stenting
Source: Kaohsiung J Med Sci. 2026 Apr 16:e70213. Online ahead of print. doi: 10.1002/kjm2.70213 (PMC13399898; doi:10.1002/kjm2.70213)

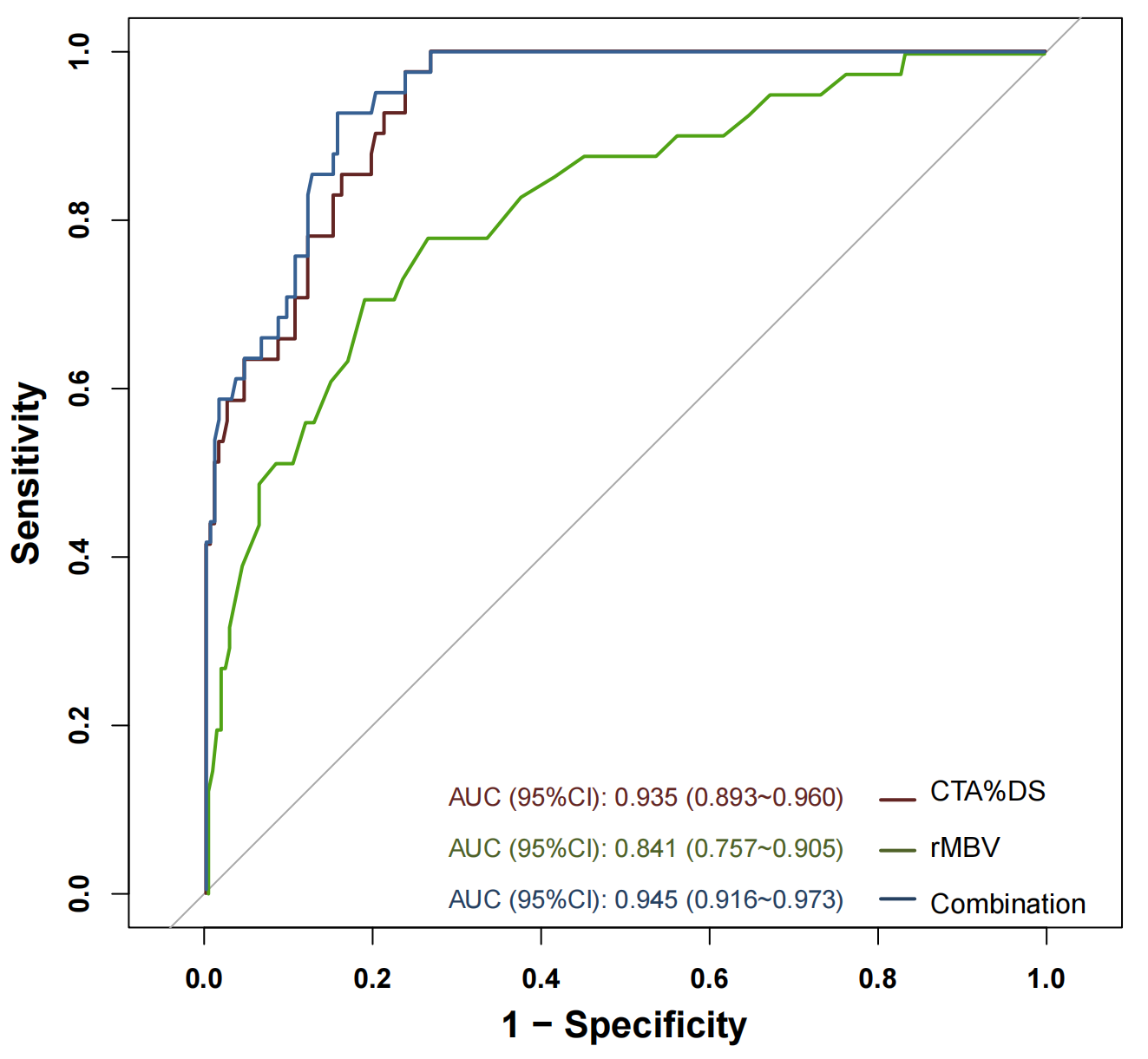

Supplement: Supplementary file 1 — Figure S1: AUC values and 95% confidence intervals (95% CI) for CTA%DS, rMBV, and their combination. [file KJM2-9999-e70213-s001.tif]
